# A pooling-LiNGAM algorithm for effective connectivity analysis of fMRI data

**DOI:** 10.3389/fncom.2014.00125

**Published:** 2014-10-06

**Authors:** Lele Xu, Tingting Fan, Xia Wu, KeWei Chen, Xiaojuan Guo, Jiacai Zhang, Li Yao

**Affiliations:** ^1^College of Information Science and Technology, Beijing Normal UniversityBeijing, China; ^2^State Key Laboratories of Transducer Technology, Shanghai Institute of Technical Physics, Chinese Academy of SciencesShanghai, China; ^3^State Key Laboratory of Cognitive Neuroscience and Learning, IDG/McGovern Institute for Brain Research, Beijing Normal UniversityBeijing, China; ^4^Center for Collaboration and Innovation in Brain and Learning Sciences, Beijing Normal UniversityBeijing, China; ^5^Department of Mathematics and Statistics, Banner Good Samaritan PET Center, Banner Alzheimer's Institute, Arizona State UniversityPhoenix, AZ, USA

**Keywords:** effective connectivity, causal structure, group analysis, functional magnetic resonance imaging (fMRI), linear non-Gaussian acyclic model (LiNGAM), pooling-LiNGAM (pLiNGAM)

## Abstract

The Independent Component Analysis (ICA)—linear non-Gaussian acyclic model (LiNGAM), an algorithm that can be used to estimate the causal relationship among non-Gaussian distributed data, has the potential value to detect the effective connectivity of human brain areas. Under the assumptions that (a): the data generating process is linear, (b) there are no unobserved confounders, and (c) data have non-Gaussian distributions, LiNGAM can be used to discover the complete causal structure of data. Previous studies reveal that the algorithm could perform well when the data points being analyzed is relatively long. However, there are too few data points in most neuroimaging recordings, especially functional magnetic resonance imaging (fMRI), to allow the algorithm to converge. Smith's study speculates a method by pooling data points across subjects may be useful to address this issue (Smith et al., 2011). Thus, this study focus on validating Smith's proposal of pooling data points across subjects for the use of LiNGAM, and this method is named as pooling-LiNGAM (pLiNGAM). Using both simulated and real fMRI data, our current study demonstrates the feasibility and efficiency of the pLiNGAM on the effective connectivity estimation.

## Introduction

Functional connectivity and effective connectivity analyses have been widely used in the neuroimaging communities (Friston, 1994; Biswal et al., 1995; Greicius et al., 2003). Functional connectivity reflects the temporal correlations between spatially remote brain regions (Friston et al., 1993), and effective connectivity evaluates the influence that one brain region exerts on others (Friston, 1994). With the ability to describe the directionality of information transferred within a brain network, effective connectivity has become a hot topic in cognitive neuroscience research.

A variety of analysis methods have been developed for estimating effective connectivity, such as the Structural Equation Modeling (McLntosh and Gonzalez Lima, 1994), Dynamic Causal Modeling (Friston et al., 2003), Granger Causality Mapping (Goebel et al., 2003), and Bayesian Network (Zheng and Rajapakse, 2006). In a number of functional magnetic resonance imaging (fMRI) effective connectivity studies, the Gaussian assumption is usually made (Geiger and Heckerman, 1994; Bollen, 1998), however, most of fMRI data possess non-Gaussion distributions. Structural Equation Modeling and Dynamic Causal Modeling are model-driven methods and may be not suitable for resting-state fMRI data (Heckerman, 2008) or for situations where the prior knowledge is insufficient. Bayesian Network is a data-driven method but requires the data to be Gaussian-distributed (Shachter and Kenley, 1989; Baker et al., 1994; Wu and Lewin, 1994). Granger Causality Mapping uses a vector autoregressive model to estimate the effective connectivity among regions. It is also data-driven and only requires the data to be wide-sense stationary and has a zero mean (Goebel et al., 2003). However, Granger Causality Mapping is sensitive to noise and down sampling, thus it may generate spurious causality under some circumstances (Geiger and Heckerman, 1994; Chen et al., 2006; Shimizu et al., 2006).

A new method named linear non-Gaussian acyclic model (LiNGAM) algorithm was proposed by Shimizu et al. (2006) and suggested to be a promising tool to estimate the causal relationship among non-Gaussian distributed data. The fundamental difference of LiNGAM from most classical effective connectivity methods is the assumption of non-Gaussian distributions. The LiNGAM algorithm utilizes higher-order distributional statistics [Independent Component Analysis (ICA)] to estimate causal relations (Shimizu et al., 2006). This algorithm is data-driven and uses the following assumptions: (a) the data generating process is linear, (b) no unobserved confounders are present, and (c) disturbance variables follow non-Gaussian distributions. With a linear, non-Gaussian setting, LiNGAM can estimate the full causal model without undetermined parameters (Shimizu and Kano, 2008), whereas methods with Gaussian data need more information to work, such as the causal ordering of variables (Shimizu et al., 2006).

The LiNGAM algorithm could perform more stably in simulated data with more data points, e.g., the number of data points ≥1000 (Smith et al., 2011). However, the number of data points is fairly small (usually no more than 300) in most fMRI experiments. One viable strategy to address this issue is to pooling data points across subjects, in this way, a larger number of data points could be submitted to the LiNGAM algorithm. In this study, this method is called as pooling-LiNGAM (pLiNGAM), and the pooling subject can be termed as the virtual subject (V-subject).

The pooling of data points from multiple subjects actually belongs to group analysis method. There are mainly three categories of group analysis techniques, including the “virtual-typical-subject” (VTS) method, the “individual-structure” (IS) method, and “common-structure” (CS) method. The VTS method assumes that every subject within a group performs the same function and has the same connectivity network, and it does not consider inter-subject variability (Li et al., 2008). The IS method learns a network for each subject separately and then performs group analysis on the individually learned networks (Goncalves et al., 2001; Li et al., 2007). It considers inter-subject variability but may not integrate group data tightly enough (Li et al., 2008). The CS method imposes the same network structure on each subject, while allowing different parameters across subjects (Mechelli et al., 2002; Kim et al., 2007). It considers the group similarity at the structural level and inter-subject variability at the parameter level (Li et al., 2008). Each technique has its own advantages. Specifically, the VTS approach fits the data when inter-subject variability is assumed minimal, for example healthy subjects; the IS approach fits the data with large inter-subject variability, such as patients with large ranged clinical scores; while the CS approach otherwise (Li et al., 2008). The pLiNGAM used in this paper belongs to the VTS technique, thus our current study only considered the case where the inter-subject variability is low, such as the healthy subjects group.

In this paper, we aimed to demonstrate the feasibility of pLiNGAM on the estimation of effective connectivity by pooling data points across subjects. First, in order to examine the validity of pLiNGAM, the simulated fMRI data that is described in Smith's study (Smith et al., 2011) was adopted. Then, to verify the practicability of pLiNGAM algorithm, the real fMRI data was further used.

## Materials and methods

### Methods

In this section, the original LiNGAM theory and the proposed pLiNGAM theory will be introduced.

#### LiNGAM theory

The LiNGAM algorithm has the following properties:

- Suppose *x*_*i*_ (*i* ∈ {1, …, *m*}, *x*_*i*_ stands for the observed variables) can be arranged in their causal order *k*(*i*). For example, as in the Gaussian Bayesian theory, there are two observed variables *x* and *y*, if *x* is the parent node of *y*, then the causal order of *x* and *y* satisfy the relation of *k*(*x*) > *k*(*y*). The generating process of variables *x*_*i*_ is recursive (Shimizu and Kano, 2008) and can be represented graphically by a directed acyclic graph (Pearl, 2000; Spirtes et al., 2000).
- Each variable *x*_*i*_ is a linear function of the preceding/parent variables, a “disturbance” term *e*_*i*_, and an optional constant term *c*_*i*_, that is
  (1) $$x_{i}=\sum_{k(j)\;<\;k(i)}b_{ij}x_{j}+e_{i}+c_{i}$$
  where *b*_*ij*_ is the weight coefficient, *k*(*i*) is the causal order for each variable.
- The disturbances *e*_*i*_ are non-Gaussian distributions, non-zero variances, and independent of each other.
  After subtracting the mean from each variable *x*_*i*_ and re-writing the equation in a matrix form, the following equation can be obtained:
  (2) x=Bx+e
  where ***x*** is data vector containing the component *x*_*i*_, ***B*** is the weight coefficients matrix and can be permuted to a strict lower triangular matrix if the causal ordering of variables is known (strict lower triangular matrix is defined as the lower triangular matrix with all zeros on the diagonal) and ***e*** is a disturbance term. Then, we can have:
  (3) x=Ae
  where ***A*** = (***I*** − ***B***)^−1^. Matrix ***A*** can be permuted to lower triangular (all diagonal elements are non-zero). For Equation (3), the independence and non-Gaussianity of ***e*** define the special ICA model.

ICA is commonly used to discover hidden sources from a set of observed data when the sources are non-Gaussian and maximally independent. In this algorithm, FastICA (Hyvärinen and Oja, 1997) is chosen to estimate the sources ***e*** and the weight coefficients matrix ***B***. However, there are two essential indeterminacies that ICA cannot solve: the order of independent components and the scaling of independent component amplitudes (Comon, 1994). In LiNGAM algorithm, the first indeterminacy can be solved by reordering the components following the rule that matrix ***B*** is a strict lower triangular matrix. If the results cannot be reordered to lower triangular, approaches have been produced to set the upper triangular elements to zero by changing the matrix as little as possible (Goebel et al., 2003). The second indeterminacy is usually handled by fixing the weights of their corresponding observed variables to unity. To assess the significance of the estimated connectivity for the LiNGAM algorithm, three statistical tests are usually performed to prune the edges of the estimated network: (a) Wald test, testing the significance of *b*_*ij*_; (b) chi-square test, examining an overall fit of the model assumptions; and c) difference chi-square test, comparing nested models (Shimizu et al., 2006).

#### pooling-LiNGAM (pLiNGAM) theory

To avoid the fatigue of subjects and ensure the quality of the data, researchers often conduct relatively short fMRI experiments. The length of time for data acquisition from these experiments is usually limited, such as 480 s (8 min), thus may result in the unstable results of LiNGAM algorithm. To address this issue, the pLiNGAM algorithm of pooling data over multiple subjects is proposed (Smith et al., 2011).

In this method, long enough fMRI data points are obtained for an artificial subject, referred to as the “V-subject,” by pooling several single subjects. As a V-subject is constructed from more than one single subject, it is preferred to assume that the inter-subject variability can be ignored. Here we provide formulated forms of extended LiNGAM, which is pLiNGAM. Suppose there are *n* subjects, then each variable ***x*** = (*x*_*i*1_, *x*_*i*2_, …, *x*_*in*_) (*i* ∈ {1, …, *m*}) is a linear function of the preceding/parent variables and a “disturbance” term ***e*** = (*e*_*i*1_,*e*_*i*2_, …, *e*_*in*_) and an optional constant term ***c*** = (*c*_*i*1_,*c*_*i*2_, …, *c*_*in*_), that is

(4) $$\begin{array}{l} \left(x_{i1},x_{i2},\ldots ,x_{in})=\sum_{k(j)\;<\;k(i)}{b^{\prime }}_{ij}(x_{j1},x_{j2},\ldots ,x_{jn}\right)\\ \;+(e_{i1},e_{i2},\ldots ,e_{in})+(c_{i1},c_{i2},\ldots ,c_{in}) \end{array}$$

where *b*^′*ij*^ is the weight coefficient, *k*(*i*) belongs to the causal order and ***e*** = (*e*_*i*1_, *e*_*i*2_, …, *e*_*in*_) is non-Gaussian distributions, non-zero variances and independent of each other.

Then the mean is subtracted from each variable *x* = (*x*_*i*1_, *x*_*i*2_, …, *x*_*in*_), the equation can be rewritten in a matrix form as:

(5) $$\begin{array}{l} \left[\begin{array}{cccc} x_{11} & x_{12} & \cdots & x_{1n}\\ \cdots & \cdots & \cdots & \cdots \\ x_{m1} & x_{m2} & \cdots & x_{mn} \end{array}\right]=\left[\begin{array}{cccc} b_{11} & b_{12} & \cdots & b_{1m}\\ \cdots & \cdots & \cdots & \cdots \\ b_{m1} & b_{m2} & \cdots & b_{mm} \end{array}\right]\\ \;\left[\begin{array}{cccc} x_{11} & x_{12} & \cdots & x_{1n}\\ \cdots & \cdots & \cdots & \cdots \\ x_{m1} & x_{m2} & \cdots & x_{mn} \end{array}\right]+\left[\begin{array}{cccc} e_{11} & e_{12} & \cdots & e_{1n}\\ \cdots & \cdots & \cdots & \cdots \\ e_{m1} & e_{m2} & \cdots & e_{mn} \end{array}\right] \end{array}$$

If we abbreviate the matrixes, (5) can be expressed as:

(6) $$x^{\prime }=B^{\prime }x^{\prime }+e^{\prime }$$

where ***x*′** denotes the variable matrix, ***B*′** is the weight coefficients matrix and can be permuted to a strict lower triangular matrix according to the causal ordering of variables. Then we can get the form of Equation (6) the same as Equation (2).

Based on the Equation (6), we can also get Equation (7) that defines the special ICA model as follows:

(7) $$x^{\prime }=A^{\prime }e^{\prime }$$

where *A*′ = (*I* − *B*′)^−1^.

**Figure 1:**
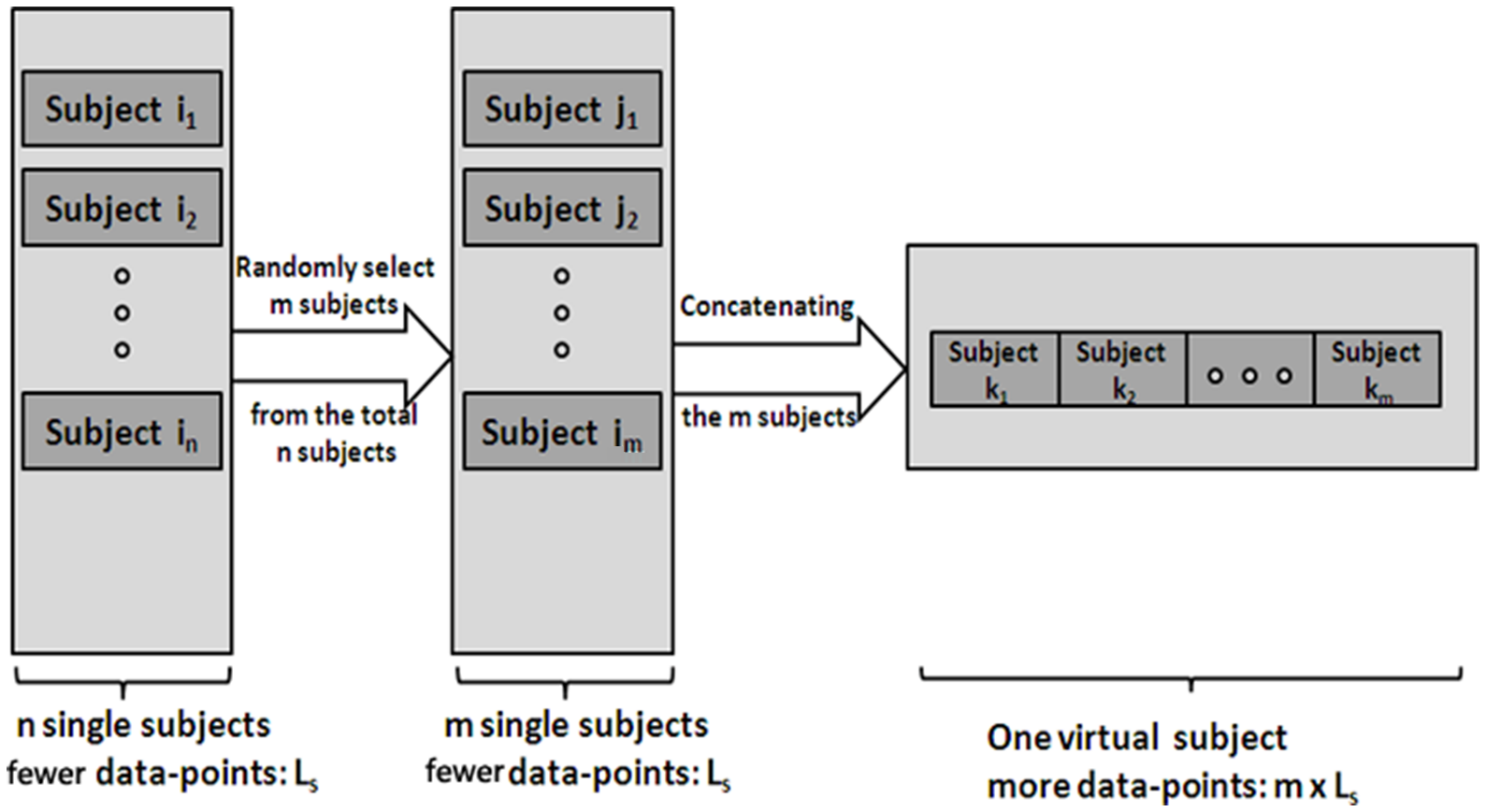
**The procedure of generating the V-subjects.** Subject *i*_1_ … *i*_*n*_ stands for the total *n* single subjects, which have few data-points. Subject *j*_1_ … *j*_*m*_ stands for the *m* subjects selected from the total *n* subjects. Then the V-subject is the pooling data of the *m* selected subjects in a random order.

The specific steps of pLiNGAM based on V-subjects consist of the following steps:

1. Generate V-subjects. First, randomly select *m* (1 ≤ *m* ≤ *n*) subjects (the length of a single subject is *L*_*s*_) from the total *n* subjects. Then, the *m* subjects' data are pooled into one V-subject with a randomly order. The length of each V-subject is therefore *L*_*m*_ = m^*^L_*s*_. Figure 1 illustrates the procedure.
2. Apply LiNGAM algorithm to the V-subjects. Default parameters of the ICA-LiNGAM algorithm are used, except for the “*skew*” instead of the “*tanh*” nonlinearity because the “*skew*” nonlinearity presents better results (Smith et al., 2011).

The error of the pLiNGAM algorithm is measured by the false positive ratio (FPR), false negative ratio (FNR), false direction ratio (FDR) and the sum of FPR, FNR, and FDR. FPR stands for the ratio of the number of falsely added edges to the whole possible existing edges, FNR denotes the ratio of the number of falsely missed edges to the whole possible existing edges, and FDR is the ratio of the number of edges that are wrongly identified in the direction to the whole possible existing edges. Furthermore, the sum of FPR, FNR and FDR is calculated to represent the total error of pLiNGAM.

### Simulated fMRI data

The simulated data are from Smith et al. in their 2011 publication (Smith et al., 2011), which have been widely used in fMRI studies (Cole et al., 2010; Smith et al., 2011). The simulations are generated using the Dynamic Causal Modeling fMRI forward model (Friston et al., 2003), in which the Dynamic Causal Modeling uses a nonlinear balloon model (Buxton et al., 1998) for the vascular dynamics. These data can provide 28 simulations, and we select the No. 7 simulation set which has 5000 data points in this paper because it has more than enough data points for the purpose of our study. The No. 7 simulation set contains 5 nodes with 250 min of data at a repetition time of 3 s. The total number of data points is 5000 (scans) for each of the 50 simulated subjects. The coefficients matrix used to generate these 50 subjects data have the same structure with slightly different coefficients.

### Real fMRI data

#### Participants

12 healthy right-handed young students, including 5 males and 7 females (mean age: 21 years) participate in our study. This study is supported by the Beijing Normal University Imaging Center. All subjects have provided written informed consent.

#### Data acquisition

Images are acquired using a Siemens Trio 3-Tesla scanner (Siemens, Erlangen, Germany) in the National Key Laboratory for Cognitive Neuroscience and Learning, Beijing Normal University. Participants are instructed to remain motionless, close their eyes but stay awake during the entire scanning procedure which lasts for 8 min. All of the functional data are acquired using an echo-planar imaging sequence with the following parameters: 33 axial slices, *TR* = 2000 ms, *TE* = 30 ms, acquisition voxel size, 3.13 × 3.13 × 3.60 mm^3^, in-plane resolution = 64×64 and matrix = 64 × 64, 240 volumes.

#### Data analyses

***Data preprocessing***. The first five volumes of the total 240 volumes in the functional fMRI data are removed to make the signal more stable. Image preprocessing including slice timing, realignment, normalization, and smoothing (FWHM = 8 mm) are conducted using the SPM8 software (http://www.fil.ion.ucl.ac.uk/spm).

**Figure 2:**
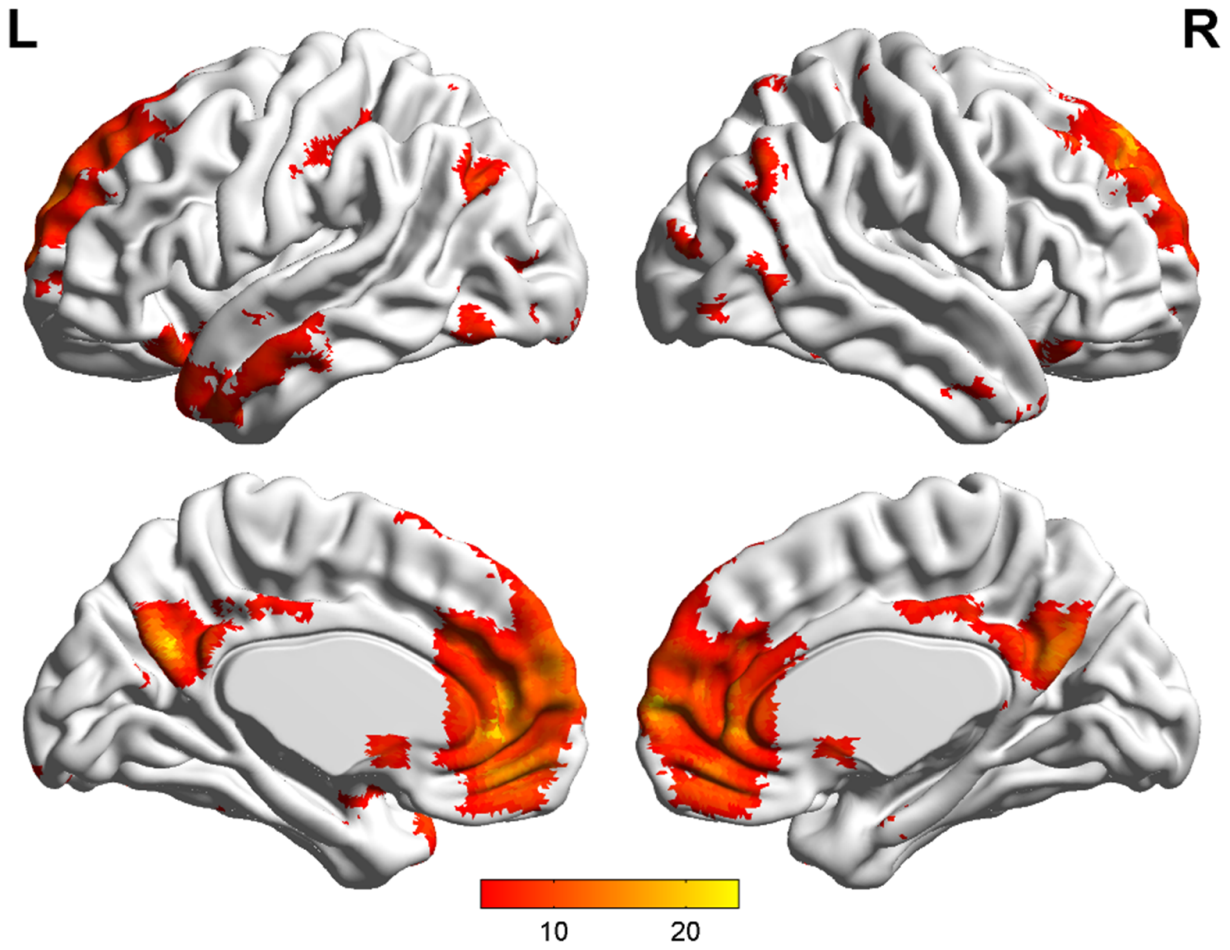
**DMN identified by group ICA (*p* < 0.05, false discovery rate corrected)**.

***Default mode network (DMN) and regions of interest (ROIs)***. Group ICA is performed to the preprocessed data using the fMRI toolbox (http://mialab.mrn.org/software/#gica) to determine the default mode network (DMN). In recent years, ICA has been widely used to identify the low-frequency neural network during resting-state or cognitively undemanding fMRI scans (Calhoun et al., 2001; Greicius and Menon, 2004; van de Ven et al., 2004). The Group ICA includes two rounds of principal component analysis, ICA separation and back-reconstruction. In ICA separation, the Extended Infomax algorithm is used (Lee et al., 1999). To select the independent component that best matches the DMN, a DMN template is developed based on a dataset of regions reported by Greicius et al. (Greicius and Menon, 2004). Subsequently, the DMN at the single subject level is acquired, and one sample *t*-test (*p* < 0.05, false discovery rate corrected) is performed (Figure 2). Figure 2 shows the regions with significant connectivity at the resting state including the medial prefrontal cortex (mPFC), posterior cingulate cortex (PCC), left/right inferior parietal cortex (lIPC/rIPC), left/right lateral and inferior temporal cortex (lITC/rITC), and left/right (para) hippocampus (lHC/rHC). Then, these eight core DMN regions are selected as nodes (ROIs) for the LiNGAM analysis. The coordinates of the eight maximally activated voxels in the core DMN ROIs are given in Table 1, and the ROIs are generated with a sphere with 6 mm-radius centered at the voxel with the maxima local *T*-value. Then, the data points of each ROI are extracted with the software rest (http://restfmri.net/forum/index.php).

**Table 1:** **The coordinates of all the ROIs for real fMRI data (*p* < 0.05, false discovery rate corrected)**.

**ROI**	**BA**	**MNI coordinate**			***T*-value**
		***x***	***y***	***z***	
PCC	23/31	0	−57	20	20.31
*m*PFC	10	−2	62	8	19.22
*l*IPC	39	−43	−67	33	9.99
*r*IPC	39	45	−60	29	7.32
*r*HC	28/35	25	−14	−23	6.47
*l*ITC	20/21	−59	−15	−16	5.50
*r*ITC	20/21	59	−12	−20	5.19
*l*HC	28/35	−22	−15	−22	4.50

BA, Brodmann's area; mPFC, medial prefrontal cortex; PCC, posterior cingulate cortex; lIPC/rIPC, left/right inferior parietal cortex; lITC/rITC, left/right lateral and inferior temporal cortex; lHC/rHC, left/right (para) hippocampus.

***pLiNGAM on the real fMRI data***. Before applying the pLiNGAM on the real fMRI data, the distribution of the V-subject obtained from the real fMRI data is examined by the One-Sample Kolmogorov–Smirnov Test. If the distribution is non-Gaussian, then the LiNGAM will be used on the V-subject to estimate the effective connectivity network among the eight core DMN ROIs.

## Results

### Simulated validation

To verify the feasibility of pLiNGAM on the estimation of effective connectivity of fMRI data, some simulation validation are performed, including the desired number of data points that is needed to make the results of LiNGAM stable, the feasibility of the pooling of data points across multiple subjects, the effectiveness of V-subjects in pLiNGAM and the influence of pooling order on pLiNGAM.

#### Desired number of data points of LiNGAM

**Figure 3:**
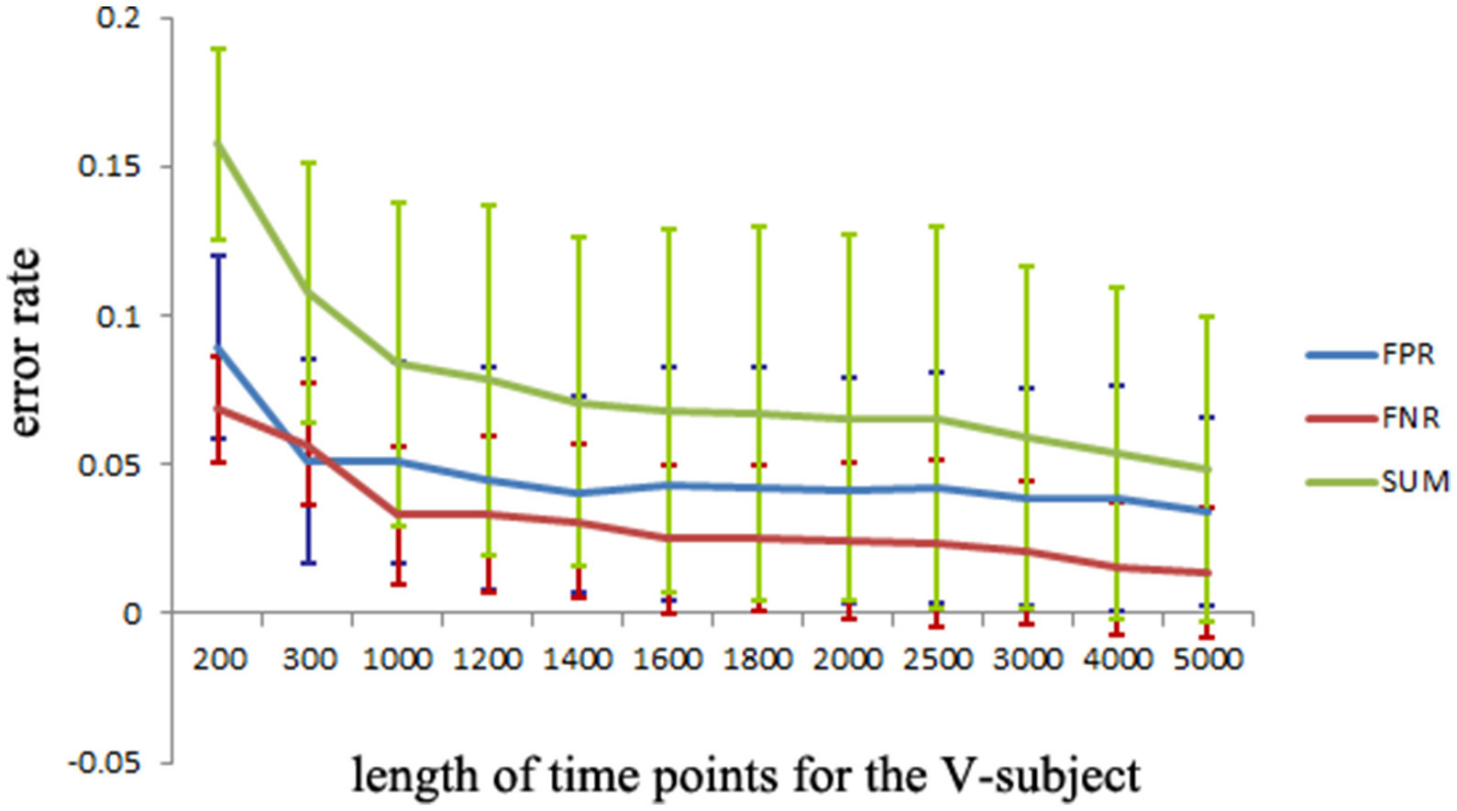
**FPR, FNR, FDR and the sum of FPR, FNR, and FDR (SUM) of the simulated subjects with different length of data points for LiNGAM algorithm (FDR is 0, so it is not shown in the figure)**.

The simulated data is used to investigate the desirable number of data points that can make the LiNGAM algorithm stable. Part of the total data points (5000 data points) of each single subject is applied to the LiNGAM. Part of data points in each subject are selected at the beginning of the total data points and the length of the points ranges from 200 to 5000. To avoid the influence of differences between subjects, the LiNGAM algorithm is applied to 50 subjects and the FPR, FNR, and FDR are calculated by averaging the fifty results. The average FPR, FNR, and FDR and the sum of FPR, FNR and FDR are shown in Figure 3 (FDR is 0, so it is not shown in the figure). Three statistical tests: Wald test, chi-square test, and difference chi-square test (*p* = 0.05) are performed to prune the edges of the estimated network. Figure 3 illustrates that both FPR and FNR are consistently decreasing as the number of data points increases. The sum of FPR and FNR reduces to approximate 7% when the length of data points arrives 5000. Because of the limitation of the number of total data points, this algorithm is not tested with longer data points.

#### Feasibility of subject pooling

To confirm pooling over subjects' data is a feasible method, the following two validations are performed.

- First, test if the pooling step could keep the distribution of the data non-Gaussian. Use the Kolmogorov–Smirnov Test to examine the distribution of the data. For the simulated data, the distribution of each single subject and several V-subjects is tested. The V-subjects were constructed as shown in Figure 1. Each of these V-subjects is pooled with several (range from 1 to 25) single subjects (each with 200 data points), then 25 V-subjects with the length of data points ranging from 200 to 5000 can be constructed. The results of the Kolmogorov–Smirnov Test in Table 2 show that all the single subjects and the V-subjects are significant non-Gaussian distribution. Furthermore, we note that the main difference of the distribution of the single subjects or V-subjects from Gaussian is the “peakedness,” then one classical measurement of the “peakedness” for non-Gaussian distribution named Kurtosis Test is adopted (Hyvärinen and Oja, 2000). The results show that the data has different kurtosis value from 3, e.g., 3.41, 3.98, 4.99 (the kurtosis value of Gaussian distribution is 3), further indicating the deviation of the data from Gaussian distribution. All these results indicate that the V-subjects are feasible to the LiNGAM algorithm.
- Second, test if the pooling step could improve the accuracy of the estimated model, in other words, test whether the result of pooling of subjects is better than that of single subject. Three groups of data are modeled: single-subject_2000 (G1), V-subject_2000 (G2), and single-subject_200 (G3). More specifically, the single-subject_2000 group consists of 10 subjects and each single subject has 2000 data points. The V-subject_2000 group is a V-subject with 2000 data points, which are pooled from 10 single subjects with 200 data points each. The single-subject_200 group consists of 10 single subjects and each single subject has 200 data points. The 10 subjects used in this paper are randomly selected from the total 50 subjects and the pooling order is random.

**Table 2:**
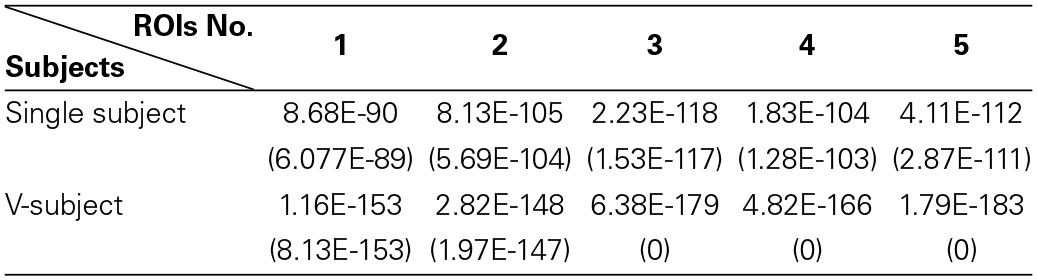
**The *p*-value [mean (STD)] of One-Sample Kolmogorov–Smirnov Test of 5 ROIs for the simulated fMRI data**.

**Figure 4:**
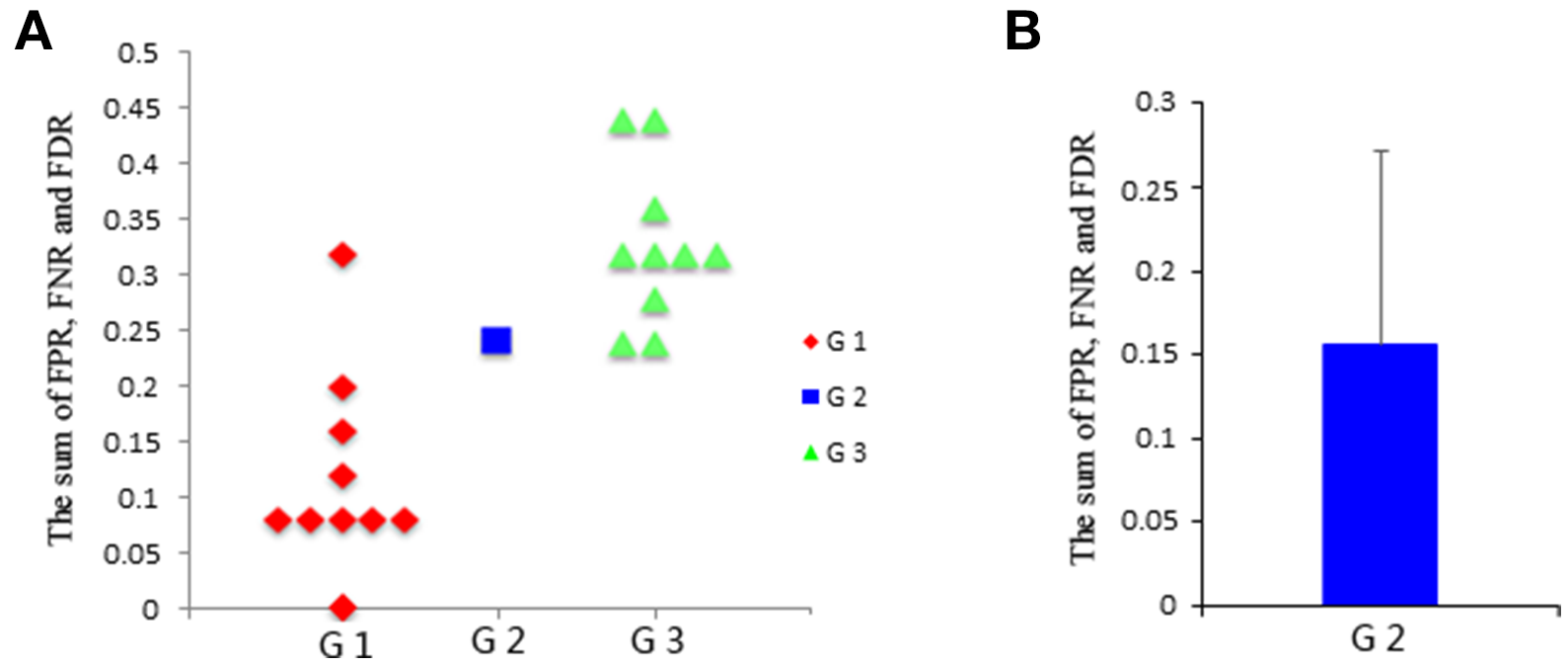
**(A)** The sum of FPR, FNR, and FDR of three groups: G1: single-subject_2000, G2: V-subject_2000, and G3: single-subject_200; **(B)** the sum [mean (SD)] of FPR, FNR, and FDR of the G2 group across 50 different V-subject_2000s.

Then, the FPR, FNR, and FDR of these three groups are calculated, and the sum of FPR, FNR, and FDR for the three groups is shown in Figure 4A. The results clearly show that the G1 group has a smaller sum of FPR, FNR, and FDR compared to the other two groups, and the G2 group has a smaller sum of FPR, FNR, and FDR than the G3 group. Furthermore, one sample *t*-test is performed on G3 and G1 respectively to verify whether the mean of G3 or G1 is significantly different from G2. The results are encouraging (*T* = −4.291, *p* = 0.002 for G1; *T* = 3.973, *p* = 0.003 for G3). These statistical results denote that the G1 group shows better results than both the G2 group and G3 group, and G2 group shows better results than G3 group, which indicating that subject pooling is feasible for the LiNGAM algorithm, and pLiNGAM can offer better results when data points were few for the single subjects. Furthermore, to test if the error rate of the G2 group is stable across different subsets of 10 single subjects, 50 V-subject_2000 are constructed by randomly selecting 10 single subjects. The sum of FPR, FNR, and FDR of these V-subject_2000 are then calculated, and the results show that the error rate of the G2 group is stable across different selections of the 10 subjects (Figure 4B).

#### pLiNGAM with V-subjects

**Figure 5:**
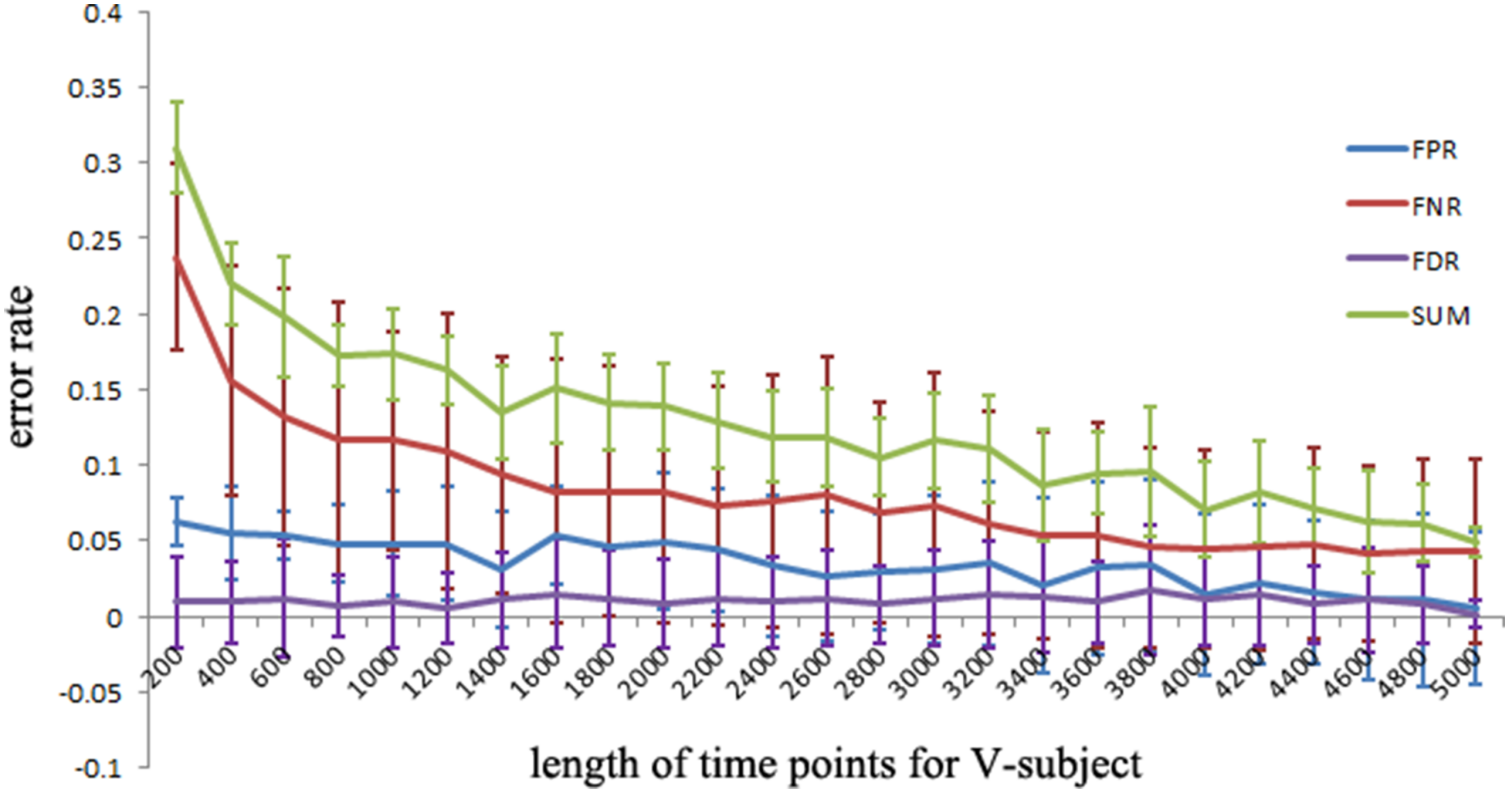
**The FPR, FNR, FDR and the sum of FPR, FNR, and FDR (SUM) of the simulated V-subjects for pLiNGAM algorithm.** The length of data points of V-subjects ranges from 200 to 5000.

To explore the FPR, FNR, and FDR estimated using the pLiNGAM with the V-subjects, the V-subjects are constructed according to the schematic shown in Figure 1. Each of the V-subjects is pooled with several (range from 1 to 25) single subjects (200 data points). For example, in each single subject, 200 data points are selected at the beginning of the total data points, then the 6 single subjects with data points of 200 are combined to form one V-subject with data points of 1200. The length of data points of each V-subject ranges from 200 to 5000. To ensure the reliability of the results, 50 V-subjects are constructed for each length of data points. Figure 5 demonstrates that when data points are more than 2000, the sum of FPR, FNR, and FDR reaches 15%, which is better than most other effective connectivity methods (Cole et al., 2010; Smith et al., 2011).

#### Influence of the pooling order

To determine whether the order of pooling subjects has any effect on the estimated network, the following test is conducted. 10 single subjects are randomly selected from the total 50 subjects. Among 3628800 possible orders, 3000 orders are randomly selected to examine this effect. For each of the 3000 pooling orders, a V-subject is generated. Then, the pLiNGAM algorithm is applied to these V-subjects and the FPR, FNR, and FDR are calculated. Our results show that the estimated network has no relation with the order of pooling, which is consistent with the fact that the major advantage of concatenation of data points across subjects in ICA is ordering the components in different subjects in the same way (Calhoun et al., 2001).

### Real fMRI validation

The distribution of the V-subject from the real fMRI data follows non-Gaussian according to the One-Sample Kolmogorov–Smirnov Test (shown in Table 3). Thus, the pLiNGAM is applicable for the real fMRI data.

**Table 3:**
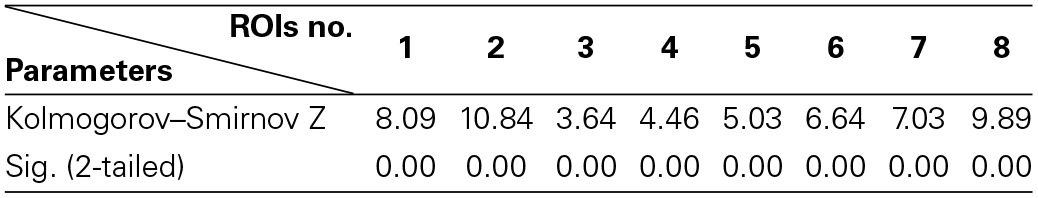
**The result of One-Sample Kolmogorov–Smirnov Test of 8 ROIs for real fMRI data**.

“Sig” represents the asymmetry significance. When the two-tailed asymptotic significance of each ROI is less than 0.05, the test distribution is not normal.

In this section, the stability of causal network is tested on the real fMRI data using pLiNGAM, and the results of effective connectivity for the real fMRI data are also displayed.

#### The stability of effective connectivity on real fMRI data

pLiNGAM is tested with different subsets of subjects from the real fMRI data to validation the robustness and stability of the result. Several subjects, *n* = 3 for example, are randomly selected from all the subjects (a total of 12 subjects) to construct the V-subject for 100 times (3, 4, 5, 6, 7, 8, and 9 subjects are tested respectively to ensure the procedure of random selection be repeated for 100 times, while 1, 2, 10, 11, 12 subjects can't be randomly selected for 100 times and are not used for testing). For each number of subjects, the causal network is analyzed for 100 times, and the common structure of the 100 causal networks is then considered as a baseline to calculate the FPR, FNR, and FDR of each causal network. Then the average of the sum of the FPR, FNR, and FDR is taken as the variability of the results. As it is shown in **Figure 7**, the variability of different subsets of the subjects is not high (about 0.26 for different number of subjects). This variability is comparable with the results of many algorithms that are mentioned in Smith et al., such as Granger, Bayes net and so on (Smith et al., 2011). Furthermore, the variability of different subsets of the subjects is stable along with the increased number of subjects (slightly decrease). These results indicate the stability and robustness of the causal networks that obtained by pLiNGAM.

#### The results of effective connectivity for real fMRI data

**Figure 6:**
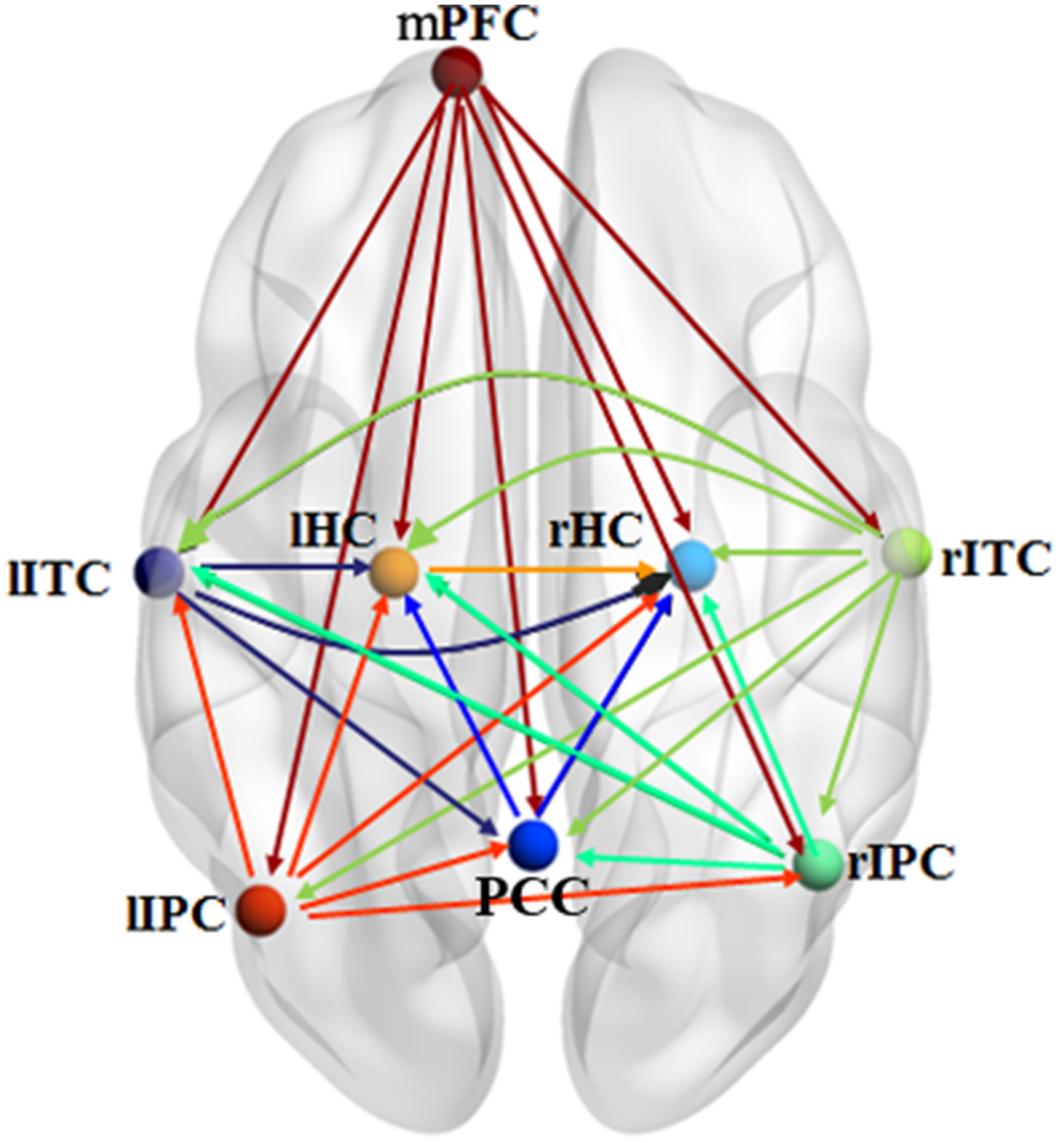
**Effective connectivity model of DMN during the resting state explored by pLiNGAM.** The different line colors indicate connections originating from different nodes. The effective connectivity has been corrected using Wald statistics, chi-square test and difference chi-square test with *p* < 0.05 as the significant level.

Figure 6 shows the effective connectivity model of DMN during the resting state investigated by the pLiNGAM algorithm (using all the 12 subjects). From Figure 6, we can conclude the following connections: *m*PFC→*r*HC/*r*IPC/*l*ITC/PCC/*r*ITC/*l*IPC/*l*HC, *r*IPC→PCC/*r*HC/*l*HC/*l*ITC, *r*ITC→*r*IPC/*l*IPC/PCC/*l*ITC/*l*HC/*r*HC, *l*ITC→PCC/*l*HC/*r*HC, *l*IPC→PCC/*r*IPC/*r*HC/*l*HC/*l*ITC (*p* < 0.05, Wald statistics). Seven direct connections are detected between *m*PFC, *r*HC, *r*ITC, *l*HC, *l*IPC, PCC, and the other ROIs. Interestingly, all links associated with *m*PFC are out-going connections, and all links associated with *r*HC are in-going connections. Furthermore, six of the total seven links associated with *r*ITC are out-going connections, and six of the total seven links associated with *l*HC are in-going connections. In addition, five of the total seven links associated with *l*IPC are out-going connections, and five of the total seven links associated with PCC are in-going connections.

## Discussion

This study employs the pLiNGAM algorithm to explore the effective connectivity of fMRI data with the V-subject. The results demonstrate that the pLiNGAM is feasible for both simulated and real fMRI data.

The pLiNGAM algorithm has several advantages in estimating the effective connectivity of brain areas. First, the simulated fMRI data demonstrate that pLiNGAM produces a more robust effective connectivity model with the V-subject than the original single subject. With a small number of data points, however, the computational stability of pLiNGAM cannot be guaranteed because in ICA estimation, the weight matrix ***B*** often converges on different values when there are not enough data points (Goebel et al., 2003). Second, this algorithm is based on the assumptions of non-Gaussianity of disturbance variables, linearity and an acyclic model, which allow the identification of the full causal model. Previous methods (Pearl, 2000; Shimizu and Kano, 2008) based on the assumption of Gaussianity require additional information (such as the causal order of variables) to obtain a full causal model (Shimizu et al., 2006). Third, a V-subject composed of more than one subject can provide more valuable information compared to a single subject. Fourth, the sum of FPR, FNR and FDR for the V-subjects can fall to 15% (Figure 5), which is smaller than most of other approaches (45%) (Cole et al., 2010; Smith et al., 2011).

Our results of the simulated data show that the sum of FPR, FNR, and FDR can just reduce to approximate 7% but not 0% when there are sufficient number of data points (shown in Figure 5), indicating that we can't obtain a perfect network of the simulated data even if the data points are long enough. This situation is explainable. A sampling step was done in the procedure of generating the simulated data (Smith et al., 2011), thus may result in the loss of information about the data. Furthermore, some noises are also added into the simulated data (Smith et al., 2011). All these process may cause the imperfect performance of pLiNGAM even when the data points are long enough.

The subject pooling has been verified to be a reasonable method through the simulated fMRI data. Then this method is applied to the real fMRI data, and the results show that the causal network is reliable and stable across different subsets of subjects, which further indicated the feasible application of pLiNGAM in the situation with low inter-subject variability. Furthermore, most of the links associated with the PCC are in-going connections, demonstrating that the PCC acts as a confluent node. Similar conclusions have been acquired in the previous studies (Li et al., 2012; Yan et al., 2013). In addition, the links associated with *m*PFC show good consistency because all links are out-going connections. Li et al.'s (2012) study also supports this result.

**Figure 7:**
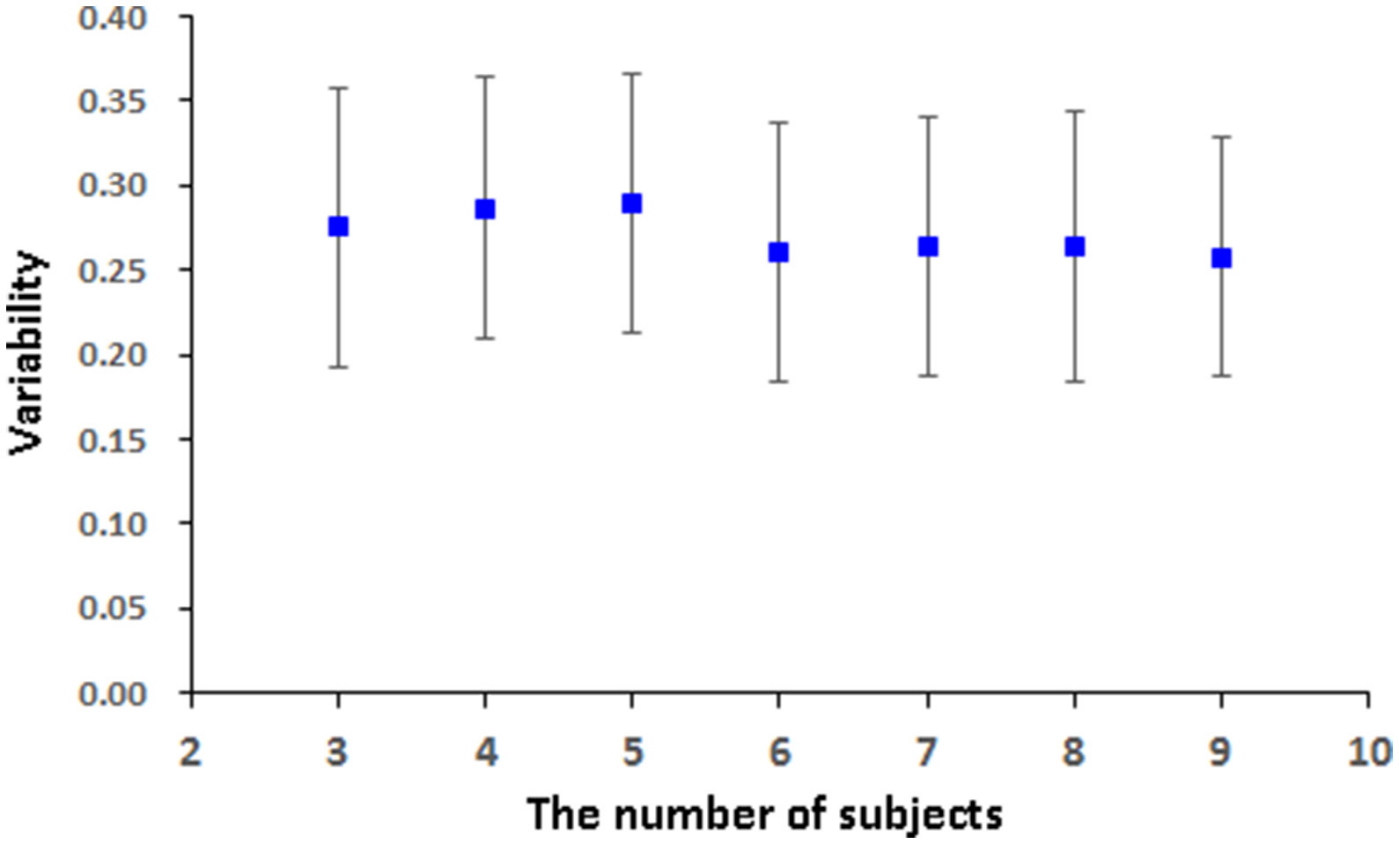
**The variability [mean (STD)] of the 100 V-subjects constructed from different number of subjects (3, 4, 5, 6, 7, 8, and 9 respectively) for the real fMRI data with pLiNGAM algorithm**.

The variability in Figure 7 for the real fMRI data is not significantly decreasing (slightly decreasing) as the number of subjects increases, which is different from the results of the V-subject in Figure 5. This may because that the variability in the real fMRI data is more stable than that of the simulated data, thus having reached the flat part toward the tail like that in Figure 5. To a certain extent, the variability is stable (slightly decrease) along with the increased number of subjects for the real fMRI data, which indicates the stability and robustness of the causal networks that obtained by pLiNGAM. In any way, further detailed explorations are needed to delve into this problem in our future study.

While having many merits, the pLiNGAM method still has several limitations. First, it only performs well when the inter-subject variability is low. pLiNGAM is one form of the “VTS” technique (Li et al., 2008), which assumes that every subject within a group performs the same function and has the same connectivity network. Other group analysis method based on LiNGAM, such as the algorithm proposed in Shimizu (2012), assumes that each subject shares a causal ordering but different connection strengths, which is similar with the “CS” approach (Li et al., 2008). So this algorithm in Shimizu (2012) may perform worse than pLiNGAM when the inter-subject variability is low (e.g., the healthy subject group), while better than pLiNGAM when inter-subject variability is a little larger (e.g., patient group). Therefore, more efforts are needed to improve pLiNGAM in order to be applicable for more general situations. Second, the V-subjects have more data points, thus may result in longer calculation time. In addition, the calculation time also depends on group sizes and the number of ROIs (Hyvärinen and Oja, 1997). Third, the assumption of an acyclic model may be a limitation to the fMRI data. This assumption implies that information can only be transmitted from one ROI to another, but not transmitted back. However, feedback is an important feature for biological systems, such as cortico-subcortical loops (Lynch and Tian, 2006). In any way, further exploration is needed to improve the pLiNGAM algorithm.

### Conflict of interest statement

The authors declare that the research was conducted in the absence of any commercial or financial relationships that could be construed as a potential conflict of interest.
